# Cervical kyphosis in asymptomatic populations: incidence, risk factors, and its relationship with health-related quality of life

**DOI:** 10.1186/s13018-019-1351-2

**Published:** 2019-10-15

**Authors:** Shuang Ao, Yu Liu, Yu Wang, Hao Zhang, Hui Leng

**Affiliations:** Department of Spinal Surgery, Chifeng Municipal Hospital, Chifeng, Inner Mongolia China

**Keywords:** Incidence, Risk factor, T1 slope, Thoracic kyphosis, Cervical kyphosis, Health-related quality of life, Asymptomatic populations

## Abstract

**Background:**

Cervical kyphosis has been pointed out in asymptomatic populations. The purposes of this study were (1) to investigate the incidence of cervical kyphosis in asymptomatic populations, (2) to identify risk factors related to cervical kyphosis, and (3) to assess the relationship between cervical kyphosis and health-related quality of life (HRQOL).

**Methods:**

A cohort of 235 asymptomatic volunteers’ records was retrospectively analyzed. Radiographic parameters of the coronal and sagittal planes were measured in the full-length spine x-ray. All patients were classified into two groups based on the cervical lordosis angle: cervical lordosis (CL) and cervical kyphosis (CK). HRQOL was evaluated by EQ-5D and SF-36 (PCS and MCS) questionnaires.

**Results:**

CK was observed in 90 of 235 (38.3%) participants. There was a significant difference with regard to age between volunteers with CK and CL (32.23 ± 8.12 vs. 42.12 ± 6.14, *p* < 0.05). Several parameters had a significant relationship with CK, including TK, T1 slope, TIA, SVA, and CT. Logistic regression analysis identified age, TK, T1 slope, and SVA as independent risk factors of CK. In addition, there was a negative correlation between CK and the parameters of HRQOL (EQ-5D, − 0.63; PCS, − 0.68; MCS, − 0.59).

**Conclusions:**

The incidence of CK in normal populations is 38.3%. Some spinal parameters are related to CK. CK is associated with the HRQOL.

## Background

It is commonly accepted that abnormal sagittal spinal alignment may contribute to the decline of health-related quality of life (HRQOL) [1, 2]. During the last decades, many published studies have revealed the important relationship between the sagittal alignment of the thoracolumbar spine and HRQOL in patients with or without spinal diseases. A variety of criteria regarding the normal thresholds of global or regional parameters for sagittal alignment have been established [3]. However, little publication reported that HRQOL deteriorates not only because of the lumbar spine and pelvic malalignment but also because of cervical deformity [4].

Cervical deformity occurs in both sagittal and coronal planes. Cervical kyphosis is the most prevalent deformity of the cervical spine. Once the onset of cervical kyphosis begins, the deformity has a tendency to perpetuate itself, with the forward shifting of the head and neck inducing abnormal forces that lead to further progression of the deformity. The prevalence of cervical kyphosis was reported to be 59.5% in patients with adolescent idiopathic scoliosis [5]. Nevertheless, the incidence of cervical kyphosis in normal populations has not been well investigated. Abnormalities of cervical alignment can be debilitating and induce adverse effects on the overall functioning and HRQOL of the patient. Furthermore, that which factors could have an influence on cervical kyphosis remains unclear.

This study aimed to (1) investigate the incidence of cervical kyphosis in normal populations, (2) identify risk factors related to cervical kyphosis, (3) determine the impact of cervical kyphosis on HRQOL.

## Methods

This study is a single-center retrospective review of a prospective database, which was approved by the institutional review board of our hospital. A total of 225 volunteers participated in our health-screening program after receiving information from the general announcement of our hospital. The inclusion criteria were the age of 10 years, available whole-spine took with the patient in a standardized standing position, and informed consent provided. Patients with a history of spinal trauma, spinal deformity and any medical condition that could affect the spine (metabolic or rheumatologic) were excluded. Study variables included C2–C7 angle (C2C7), C0–C2 angle (C0C2), C2–C7 sagittal vertical axis (SVA_c2c7_), gender, age, height, weight, BMI, thoracic kyphosis (TK), lumbar lordosis (LL), sagittal vertical axis (SVA), vertical distance between C7 plumb line and center sacral vertical line (C7PL-CSVL), T1 slope [6], thoracic inlet angle (TIA), sacral slope (SS), sacral slanting, cervical tilting (CT), K-line tilt [7], pelvic incidence (PI), and lumbar pelvic relationship (LPR) [8]. The definition of the above radiographic parameters is shown in Table 1. Negative values indicated lordosis, and positive values indicated kyphosis. All patients were divided into two groups based on C2–C7 angle: the cervical lordosis group (CL; C2–C7 angle < 0°) and the cervical kyphosis group (CK; C2–C7 angle ≥ 0°). The EuroQOL five dimensions questionnaire (EQ-5D) and the Short-Form 36 (SF-36) questionnaire (mental component score (MCS) and physical component score (PCS)) were used to assess HRQOL [9, 10].

**Table 1:** The definition of the radiographic parameters

Parameter	Definition
C2–C7 angle	Angle between the lower plate of C2 and the lower plate of C7
C0–C2 angle	Angle between the McRae line and the lower plate of C2
SVA_c2c7_	The horizontal offset from the posterosuperior corner of C7 to the vertebral body of C2
TK	Angle between the superior plate of T1 and the lower plate of T12
LL	Angle between the superior plate of L1 and the superior plate of S1
SVA	The horizontal offset from the posterosuperior corner of S1 to the vertebral body of C7
C7PL-CSVL	Vertical distance between the C7 plumb line and center sacral vertical line
T1 slope	Angle between a horizontal line and the superior endplate of T1
TIA	Angle formed by a line perpendicular to the superior endplate of T1 and a line connecting the center of the T1 upper endplate and the upper end of the sternum
SS	Angle between the horizontal and the sacral plate
Sacral slanting	The angle between the horizontal line and the upper end plate of the sacrum
CT	The angle formed between the vertical line from the center of the upper endplate of T1 and the line from the center of the upper endplate of T1 to the center of C2 vertebra
K-line tilt	The angle between the K-line and a line perpendicular to the horizon
PI	Angle formed by a line perpendicular to the superior endplate of S1 and a line connecting the center of the S1 upper end plate and the caput femoris
LPR	The angle formed between a line drawn on the inferior endplate of the last vertebra in the lumbar curve and the iliac crest line

### Statistical analysis

SPSS version 20.0 (SPSS, IBM, USA) was used for all statistical analyses. The incidences reported by two previous studies were 33.9% and 41.7%, the mean of which was 37.8%. A sample size of 196 produces a two-sided 95% confidence interval with a two-sided width equal to 0.140 when the sample proportion is 0.378. Results were presented as mean ± standard deviation. The data were checked for normality and equal variances. Student’s *t* test or Wilcoxon rank-sum test was utilized to compare group differences for quantitative variables. Pearson *χ*^2^ test or Fisher’s exact test was utilized to compare categorical variables. A logistic regression model was conducted to identify independent risk factors of CK. Statistical significance was set at *p* < 0.05.

## Results

A total of 235 volunteers’ records were retrospectively reviewed in this study. Ninety (38.3%) of 235 volunteers were observed to have CK, whereas CL was found in the remaining volunteers (61.7%). The information of the two groups is shown in Table 2. Age showed a significant difference between patients with CK and CL. The remaining demographic parameters including gender, height, weight, and BMI had no difference between the two groups. Since aging plays a crucial role in the change of cervical sagittal alignment, we divided all volunteers to four groups according to age (< 25, 25–39, 40–54, and ≥ 55) as shown in Table 3. C2C7 showed a significant decrease with age. The percentage of CK decreased from 49.3 to 11.1% with the rise of age. The incidence of CK in patients ≥ 55 years was significantly decreased compared with those <55 years (*p* < 0.05). C0C2 and SVA_c2c7_ demonstrated no difference among age.

**Table 2:** Demographic data of the study cohort

	CK	CL	*p*
Number	90	145	–
Gender (F:M)	41:49	75:80	n.s.
Age (years)	32.23 ± 8.12	42.12 ± 6.14	< 0.05
Height	165.23 ± 11.34	165.71 ± 12.31	n.s.
Weight	55.34 ± 12.12	56.91 ± 11.23	n.s.
BMI	20.32 ± 1.78	20.89 ± 1.57	n.s.
C2C7	11.92 ± 6.12	−7.23 ± 5.32	< 0.05
Percentage (CK)	38.3%	61.7%	–

*F* female, *M* male, BMI body mass index

**Table 3:** The distribution of cervical sagittal parameters according to age

Age	Number	C2C7	C0C2	SVA_c2c7_	Percentage (CK)
< 25	71	8.43 ± 7.23	18.21 ± 8.32	25.21 ± 16.23	35 (49.3%)
25–39	65	5.21 ± 8.22	18.91 ± 7.45	26.32 ± 14.98	29 (44.6%)
40–54	54	1.41 ± 7.11	19.12 ± 8.34	24.89 ± 16.39	21 (38.9%)
> 55	45	− 3.23 ± 9.89	18.23 ± 7.91	26.91 ± 15.31	5 (11.1%)
*p*	–	< 0.05	n.s.	n.s.	< 0.05

The correlation coefficients between spinal parameters are shown in Table 4. C2C7 showed a significant correlation with TK (− 0.63), T1 slope (− 0.43), TIA (− 0.51), SVA (0.32), and CT (− 0.695). There was a linear correlation between TK and LL (0.43), T1 slope and TK (0.34), TIA and T1 slope (0.68), CT and TIA (0.49), CT and T1 slope (0.56), SS and LL (0.83), LL and PI (0.57), and PI and SS (0.73). Furthermore, a multiple logistic regression analysis was performed to identify independent risk factors of CK in normal populations. The results revealed that age, TK, T1 slope, and SVA were independent risk factors of CK (Table 5). No statistically significant change in the odds was observed for the remaining parameters.

**Table 4:** Correlation coefficients of study parameters

	C2C7	TK	LL	T1 slope	TIA	SVA	C7PL-CSVL	CT	SS	LPR	K-line tilt	PI
C2C7	–	− 0.63*	− 0.03	− 0.43*	−0.51*	0.32*	0.10	− 0.695*	− 0.05	0.02	0.02	− 0.03
TK		–	0.43*	0.34*	0.13	− 0.08	0.13	0.15	0.21	0.13	0.04	0.21
LL			–	0.24	− 0.09	0.15	0.23	0.19	0.83*	− 0.17	0.19	0.57*
T1 slope				–	0.68*	0.11	− 0.08	0.49*	0.19	0.05	− 0.04	0.23
TIA					–	0.25	0.16	0.56*	− 0.02	0.14	0.12	0.09
SVA						–	− 0.12	0.18	0.06	− 0.22	− 0.11	− 0.18
C7PL-CSVL							–	0.05	0.02	0.23	0.14	0.16
CT								–	0.17	0.15	− 0.16	0.19
SS									–	− 0.17	0.21	0.73*
LPR										–	0.08	− 0.07
K-line tilt											–	0.14
PI												–

*p < 0.05

**Table 5:** Independent risk factors identified by logistic regression analysis

Variables	OR (95% CI)	*p*
Age	1.09 (1.03–1.15)	< 0.05
TK	0.96 (0.92–0.98)	< 0.05
T1 slope	0.97 (0.94–0.99)	< 0.05
Sacral slanting	1.07 (1.05–1.09)	< 0.05
SVA	1.05 (1.02–1.09)	< 0.05

As shown in Table 6, EQ-5D demonstrated a markedly decrease in volunteers with CK compared with participants with CL (0.72 ± 0.13 vs. 0.86 ± 0.14, *p* < 0.01). There was a significant difference regarding MCS and PCS of SF-36 between participants with CK and CL (MCS, 26.89 ± 11.78 vs. 31.18 ± 12.34; PCS, 35.24 ± 12.87 vs. 40.45 ± 11.78). Correlation analysis showed that EQ-5D, PCS, and MCS had a significant correlation with C2C7.

**Table 6:** The relationship between CK and HRQOL

	CK	CL	*p*	Correlation coefficient (C2C7)
EQ-5D	0.72 ± 0.13	0.86 ± 0.14	< 0.05	− 0.63*
SF-36				
MCS	26.89 ± 11.78	31.18 ± 12.34	< 0.05	− 0.59*
PCS	35.24 ± 12.87	40.45 ± 11.78	< 0.05	− 0.68*

*p < 0.05

## Discussion

The spinal sagittal alignment plays an important role in degenerative diseases, spinal deformity, surgical planning, and postoperative recovery [11, 12]. The majority of researches have paid close attention to lumbosacral alignment. The pelvic incidence (PI), a constant lumbosacral parameter, has a significant influence on lumbar lordosis and thoracic kyphosis, which has been widely accepted [13, 14]. Recent studies have demonstrated the significance of the cervical sagittal alignment. Mounting evidence revealed that neck pain and functional disability could be caused by disc degeneration, loss of cervical lordosis, and the inflammation of soft tissue, besides trauma, tumor, etc. [15]. However, there is a lack of large-scale study focusing on the incidence and risk factors of CK in asymptomatic populations and its relationship with HRQOL.

This study reported a 38.3% (90/235) incidence of CK in asymptomatic volunteers. To our knowledge, the study about the incidence of CK in normal populations is little. Iorio et al. reported that the overall rate of CK was 33.9% [16]. Hiyama and his coworkers have reported a small-scale investigation including 24 normal adolescents and found 10 (41.7%) adolescents having CK [5]. The incidence of CK in patients with adolescent idiopathic scoliosis (AIS) was well studied and reported to be 40–86% [5, 17–19]. AIS was a three-dimensional deformity of the spine that includes a coronal curve, vertebral rotation, and flattening of the sagittal profile [20–23]. Many studies have demonstrated that some abnormal parameters, such as TK and SVA increased the onset of CK in patients with AIS [5, 18, 24, 25]. Therefore, we hypothesized that these parameters could be related to CK in asymptomatic populations.

In the present study, we identified some factors correlated with CK, including age, TK, T1 slope, TIA, CT, and SVA. Aging is a key predictor for cervical sagittal alignment. Our study suggested that the incidence of CK in asymptomatic subjects decreased with age, which is consistent with the previous findings. CK in patients ≥ 55 years was significantly decreased compared with those < 55 years (*p* < 0.05). In the study by Iorio et al., they found that C2–C7 lordosis and C0–C7 lordosis had a significant increase with age [16]. Cervical kyphosis was present in approximately half of subjects in the < 35-, 35- to 44-, and 45- to 54-year age groups (56.7%, 50.0%, and 47.1%, respectively) compared with 9.5% of subjects between 55 and 64 years and 12.5% of those ≥ 65 years. Younger patients had a significantly higher rate of cervical kyphosis compared with older patients. Chen et al. reported that C2–C7 lordosis was significantly greater in patients ≥ 65 years than in those < 60 years [26]. Above findings are in line with the study of Park et al., who found that C2–C7 lordosis was greater in subjects > 60 compared with those < 30 years [27].

CK was negatively associated with TK in this study. Zeng et al. revealed a positive relationship (*r* = 0.272) between CL and TK, which was similar to our finding [28]. In the study by Abelin et al., C2–C7 kyphosis was negatively related to TK (*r* = − 0.510). However, Hiyama et al. found that CK had a negative correlation with TK in patients with AIS instead of normal populations [5]. Lee et al. reported that TK had a negative correlation with C0–C7 kyphosis and no relationship with C2–C7 kyphosis [29]. In addition, the negative relationship between CK and TK was observed in patients with AIS in several previous publications [2, 18, 24, 25]. Further studies should deeply investigate the effect of TK on CK in asymptomatic populations. T1 slope and TIA are T1 vertebra-related spinal parameters. There was a significant correlation between T1 slope and TIA, which is similar to published studies [29, 30]. They showed a marked relationship with C2–C7 kyphosis. In the study by Lee et al. and Xing et al., semblable results were observed [29, 30]. These findings implied that CK was influenced by not only TK but also TIA and TI slope.

Cervical sagittal alignment, as a part of global sagittal alignment, may have an effect on HRQOL [2, 31, 32]. CK, which is considered as pathologic, may be associated with the development of cervical myelopathy and neck pain [31, 33]. However, the established relationship between CK and HRQOL is lacking. In our study, we found that volunteers with CK had poor HRQOL compared with those without CK. Postoperative C2–C7 kyphosis was significantly correlated with HRQOL in patients with AIS [2]. In the study by Shin et al., females with cervical deformity had the poorest HRQOL among four groups. Meanwhile, EQ-5D was substantially lower in males with cervical deformity than in those without cervical deformity. EQ-5D showed markedly correlation with C2 and C7 SVA in their study.

There are several limitations to this study. First, all enrolled volunteers were Chinese. Therefore, it remains unclear whether these data can be applied to other races. Second, the retrospective analysis may result in some unpredictable bias for this study. Third, other radiological measurements (e.g., global coronal balance and pelvic tilt) that could affect HRQOL were not included. Last, a large-scale and multicenter study is our next proposal.

## Conclusions

This study reports a 38.3% incidence of CK and identifies some independent risk factors of CK, including age, T1 slope, SVA, and TK in asymptomatic populations. Furthermore, our study indicates that CK had an adverse effect on HRQOL.

## Data Availability

The datasets used and/or analyzed during the current study are available from the corresponding author on reasonable request.

## Abbreviations

AIS: Adolescent idiopathic scoliosis
BMI: Body mass index
C0C2: C0–C2 angle
C2C7: C2–C7 angle
C7PL-CSVL: Vertical distance between C7 plumb line and center sacral vertical line
CK: Cervical kyphosis
CL: Cervical lordosis
CT: Cervical tilting
HRQOL: Health-related quality of life
LPR: Lumbar pelvic relationship
MCS: Mental component score
PCS: Physical component score
PI: Pelvic incidence
SS: Sacral slope
SVA: Sagittal vertical axis
SVA_c2c7_: C2–C7 sagittal vertical axis
TIA: Thoracic inlet angle
